# Effective strategies for teachers to guide children’s art activities: based on a survey of children’s art activities and teacher guidance, and an analysis of their correlation

**DOI:** 10.3389/fpsyg.2025.1600401

**Published:** 2025-07-31

**Authors:** Han Zhang, Yifan Yang, Jialin Li

**Affiliations:** ^1^College of Distance Education, Shaanxi Normal University, Xi’an, China; ^2^Luanzhen Street Center Kindergarten, Xi’an, China; ^3^Jinniu District Organ in the Second Kindergarten, Chengdu, China

**Keywords:** children’s art activities, teacher’s guidance, art activity styles, art education quality, teacher instructional strategies

## Abstract

Providing effective strategic guidance for teachers to guide children’s art activities and improving the quality and level of children’s art activities is of great significance for children’s mental development and subsequent development, as well as improving the quality of teacher teaching. We developed a children’s art activity and teacher guidance observation table to collect art activity data from 538 children and guidance behavior data from 62 kindergarten teachers in 10 kindergartens in Chongqing, Sichuan, Hubei, Jiangxi, Jiangsu, and Shanghai. Then, we analyzed the differences in children’s art activities and discussed the correlation between children’s art activities and teachers’ guidance. The results showed that there are significant and extremely significant differences in the art activity styles and interests of children in random activities, independent activities, and organized activities. There is a significant correlation between teacher guidance and children’s art interaction styles. Based on the above results, we suggest that when instructing children’s art activities, teachers should build a hierarchical and interesting learning environment to stimulate children’s interest in learning art and make it fun, to continuously improve the quality and level of children’s art activities.

## Introduction

Children’s art activities are mainly under the guidance of teachers to stimulate children’s observation, association, imitation sensory contact, etc., and to show their cognitive process of art through language, movement (See and Kokotsaki, 2016). In this process, children can improve their aesthetic power and creativity, and both their emotions and personalities can be developed to a certain extent. The results of relevant studies have shown that the high quality of the whole process of children’s art activities is also the high quality of their learning results, and can promote the quality of children’s learning (Wang and Zhang, 2023). High-quality arts activities for children are strongly associated with high-quality learning outcomes and contribute significantly to the overall quality of children’s learning. Research has shown that such activities promote cognitive, emotional, and social development. For instance, in shadow theatre activities, multi-sensory participation and creative self-expression enhance imagination, self-confidence, and communication skills, supporting all-round development (Xiao et al., 2023). Liu (2024b) further found that elements like self-monitoring, concentration, and cooperation in art activities directly improve learning efficiency and social skills. On a neurological level, Baker (2025) highlighted that visual self-expression activates brain reward systems, helping children regulate emotions, manage stress, and engage more effectively in learning and social situations. Additionally, Boster et al. (2021) demonstrated that collaborative art activities can foster positive interactions among children with complex communication needs, emphasizing the role of the arts in enhancing social development.

As early as the end of the last century, the American Arts Education Partnership (AEP) began to pay attention to the research on children’s art activities, and jointly established a children’s art education system with the state art education committees. There are fewer specific studies on children’s art activities in China. Some scholars believe that the formation of children’s early sense of beauty and responsiveness to artistic phenomena is mainly influenced by the senses, intuition, and experience (Campbell, 2018; Redies, 2015). Most kindergarten teachers have limited knowledge of children’s art activities, and usually use the traditional way of instilling knowledge or letting children imitate mechanically, which leads to unsatisfactory results in children’s art education (Li, 2022). Western approaches like Reggio Emilia and the Teaching for Artistic Behavior (TAB) framework prioritize child agency, open-ended exploration, and process over product (Douglas and Jaquith, 2018). This divergence suggests cultural variations in how teacher guidance manifests (Ellis, 2022; Manera, 2022).

The two main elements of children’s art activities are activity mode and activity interest. Activity mode is the foundation of children’s art activities, belonging to the basic behaviors of children in the process of art activities, representing a child’s knowledge and enjoyment of art activities. Generally speaking, children’s curiosity is relatively strong, if the teacher correctly guides them, it is easier to stimulate their interest (Liu, 2024a). Therefore, in the process of guiding children, teachers need to help children construct methods and action plans for art learning, and through their own words, deeds, and moral cultivation, have a subtle influence on children.

Based on the above questions, we compiled an observation form to investigate and analyze changes in children’s art activities as well as in teachers’ guidance, to provide teachers with practical bases and effective suggestions for guiding children’s art activities. This study integrates Vygotsky’s sociocultural theory, which positions teacher guidance as scaffolding within the Zone of Proximal Development (ZPD), with Piaget’s constructivist view of art as a vehicle for symbolic representation (Papavlasopoulou et al., 2019; Vygotsky, 1978). Kolb’s experiential learning cycle further informs our analysis of how children engage in art through concrete creation, reflective observation, and abstract conceptualization (Quibrantar and Ezezika, 2023).

## Research design

### Development of research instruments

Drawing on rootedness theory, this study developed the observation form used in the survey. In the first step, we first investigated 62 female teachers’ usual art activity instruction cases. In the second step, the collected teaching cases were organized and analyzed to record 96 keywords related to children’s interest in art activities, children’s art activity styles, and teachers’ guidance. The third step is to further organize and categorize the keywords, leaving 41 keywords with a high frequency of occurrence. In the fourth step, the preliminary keyword list was determined, and the keywords in the word list were used as the preliminary observation items of this study, defining the connotation, behavioral description, and relevant examples of each observation item. We made a secondary determination of the observation items for children’s art activities and gave them to the relevant experts for judging. According to the guidance given by the experts, the keywords in the observation table were further organized, and the observation table officially adopted in this study was finally determined. As shown in Table 1.

**Table 1:** Number of items in each observation table and specific observations.

Observation items	Number of observation items	Specific observation items
Children’s art activity method observation form	12	Artistic experience, imitation action, classroom discussion, association, questioning, comparison, prediction, performance, description, collaborative division of labor, choice, listening
Children’s art activity interest observation form	5	Level of focus, active questioning, active participation, critical thinking, mood or emotional well-being
Teacher guidance observation form	4	Demonstration, material provision, explanation, documentation

The Children’s art activity method observation form has a total of 12 observations, each of which is defined as follows: (1) Artistic experience: Children’s direct sensory experience in participating in art activities. (2) Imitation actions: Children use their observation to imitate the behavior of classmates or teachers in art activities. (3) Classroom discussion: Children interact with classmates or teachers about an art activity topic that has multiple people interested. (4) Association: Children recall related phenomena or things through a phenomenon or thing in an art activity. (5) Questioning: In the specific context of an art activity, children ask questions and want the teacher or classmates to answer them. (6) Comparison: Children actively compare similarities and differences between multiple phenomena or things in art activities. (7) Prediction: Children try to understand the developmental process of an art activity by making predictions about the next step in the development of the phenomenon or thing based on their prior experience with the activity. (8) Performance: Children act out the characters or phenomena involved in an arts activity to attract the attention of classmates or teachers. (9) Description: Children describe the phenomenon or event in their way. (10) Collaborative division of labor: Children and their partners take on their tasks for the same activity, and work together to complete a particular task in the activity by cooperating and complementing each other. (11) Choice: When multiple things, situations, or phenomena are presented in an art activity, children spontaneously select the optimal object. (12) Listening: Children listen to the verbal expressions of their classmates or teachers in an attempt to memorize what they are saying.

The children’s art activity interest observation form has a total of five observation items each of which is defined below: (1) Level of focus: Children can concentrate on art activities and their eyes do not wander. (2) Active questioning: Children can ask questions if they question the behavior of their classmates or teachers. (3) Active participation: Children are very active in art activities and will take the initiative to help others or assist the teacher in facilitating the activity. (4) Critical thinking: Children can think about the phenomena they encounter in art activities to gain a deeper understanding. (5) Mood or emotional well-being: Children’s expressions and movements during art activities are cheerful, such as active behavior and bright eyes.

The teacher guidance observation form has a total of four observation items, each of which is defined below: (1) Demonstration: Teachers can make use of their actions and language, etc., to give an example to the students, to facilitate the children’s imitation. (2) Material provision: Teachers can take the initiative to provide certain materials, such as teaching aids, related books, audio-visual products, etc., to enable children to better participate in art activities. (3) Explanation: In the process of participating in children’s art activities, teachers can tell a certain phenomenon or thing through language to facilitate children’s understanding. (4) Documentation: Teachers record the performance and status of children’s participation in art activities through audio recording, text recording, video recording, or photo recording. Demonstration and explanation are direct guidance methods, material provision is an intermediary method, and recording emphasizes objective documentation.

### Objects of study

We observed 538 children and 62 teachers in 10 kindergartens in Shanghai, Jiangsu, Hubei, Sichuan, and Jiangxi Provinces. The total number of boys is 275 and the total number of girls is 263. The 62 kindergarten teachers are all women, with an average of eight years of teaching experience.

### Activity type classification

According to the actual situation of children’s participation in art activities in kindergartens in different provinces and districts, children’s art activities are divided into three types: spontaneous activities, self-directed activities, and structured activities.

### Spontaneous activities

Spontaneous activities refer to art activities that are naturally triggered by children’s immediate interests, sudden situations, or occasional events in kindergarten daily activities without prior planning and design. This kind of activity is usually characterized by temporary, chance and spontaneity (Moreno Córdoba, 2022). For example, during outdoor activities, children find leaves falling on the ground by chance and spontaneously collect them to create leaf collages; or during free play time, a child hums a song that he has composed, which triggers other children to participate in creating and singing the song together. In spontaneous art activities, the teacher’s role is more of an observer and supporter, providing materials, encouragement, and guidance at the right time without interfering with children’s independent exploration.

### Self-directed activities

Self-directed activities are art activities that children initiate, plan, and implement independently based on their interests, wishes, and needs. The theme, form, choice of materials, and the process of the activity are all decided by the children independently, emphasizing the subjectivity and autonomy of the children (Jamison and Kirova, 2025). For example, children decide independently to carry out creative mask-making activities in the arts and crafts area, from designing mask styles, choosing materials such as colored paper, feathers, sequins, and so on, to completing the mask-making, the whole process is completely done by children independent of themselves. In the performance area, children organize a fairy tale drama performance on their own, assigning roles and designing lines and movements. Teachers mainly play the role of guides and resource providers in independent art activities, and support children to carry out art activities in depth by creating a rich activity environment, providing diversified materials, and inspiring questions at the right time.

### Structured activities

Structured activities are carefully designed and organized by teachers according to teaching objectives, children’s age characteristics, and developmental needs. These activities include clear teaching goals, activity plans, and structural processes (Županić Benić and Mendeš, 2024). For example, the teacher organizes painting activities for children around the color theme of spring. Before the activity, the teacher gives a demonstration and guides children to observe the scenery of spring. Then the children are instructed to use painting tools to express spring or conduct musical activities to recognize musical instruments. Teachers guide children to recognize and feel musical instruments by displaying different musical instruments, introducing the characteristics of musical instruments, and playing audio of musical instruments. In structured art activities, the teacher is the structure, guide, and evaluator of the activity, leading the process and direction of the activity and ensuring that the goals of the activity are achieved.

### Research procedure

This study employed non-participant observation to objectively analyze female teachers’ art guidance behaviors and children’s responses, avoiding interference with activities. Non-participant observation was chosen over participatory methods due to its capacity to minimize observer bias while maintaining ecological validity, despite limitations in accessing participants’ internal states. Observers—master’s-trained preschool educators—underwent five days of intensive training covering early childhood art education theories, observation methodologies, and tool application. Training included simulated exercises using teaching videos and real-world practice to refine data recording skills. A hybrid sampling approach combined time sampling and event sampling to track three activity types: spontaneous activities, self-directed activities, and structured activities. Each type of activity was observed for 1 h each, totaling 3 h in length. Teachers followed standard protocols without altering routines, while researchers strictly avoided interaction to ensure neutrality. The collected data were entered into tools such as Excel and SPSS 20.0 for further processing and analysis. Qualitative data are also retained in the form of videos, photographs, texts, etc. to complement quantitative analyses.

### Validity and reliability verification of the observation form

#### Content validity

To enhance the rigor of content validity verification, we integrated both qualitative expert feedback and quantitative assessment as follows. Five experts in the field of preschool education—including three university professors and two provincial early childhood education teachers and researchers—were invited to review the content of the observation form. From the professional perspective of art activity guidance, the experts first provided open-ended suggestions on the alignment between the observation form’s indicators and the actual development of young children’s art activities, resulting in 12 revision recommendations. To complement this qualitative input, the experts were additionally asked to conduct a structured scoring task: they rated the relevance to practical art activities of each indicator on a 5-point Likert scale (1 = not relevant at all, 5 = highly relevant). The inter-rater consistency of these ratings was then analyzed using Kendall’s coefficient of concordance (W). The calculated W value was 0.78 (≥0.7 indicates high consensus among experts), confirming that the experts held consistent views on the indicators’ alignment with real-world art activities. Based on both the qualitative suggestions and this quantitative consistency check, the research team revised the observation form to ensure it comprehensively and accurately covers the key elements of early childhood art activities, thereby strengthening its content validity.

#### Reliability verification

##### Internal consistency reliability

Calculated for 62 valid observations, the Cronbach’s alpha value was 0.89, indicating high internal consistency among the observation form’s items and stable, reliable measurement results.

#### Inter-rater reliability

The raters in this study were master’s degree students majoring in preschool education. Before formal data collection, 15 raters participated in a 3-day systematic training program covering the use of the observation form, definitions of various art activity types, and norms for observational recording. Raters also practiced simulated observations and scoring, with focused discussions and explanations provided to resolve disagreements. Post-training, to test inter-rater consistency, 20 art activity cases were selected to represent diverse scenarios, specifically comprising 5 painting activities (2 watercolor, 2 wax crayon, 1 collage); 5 handicraft activities (3 paper-based, 2 clay-based); 5 music activities (2 singing, 2 percussion, 1 music game); and 5 integrated art activities (e.g., story drawing + craft decoration, nursery rhyme performance + simple prop making).

The intragroup correlation coefficient (ICC) was used for statistical analysis, yielding an ICC value of 0.91. This high consistency across diverse art activity types, organizational forms (individual/group), and difficulty levels (simple/complex material use) confirms strong inter-rater reliability and robustness of the scoring results across varied contexts.

## Research findings

### Comparison of the variability of children’s art activity styles across types of activities

The study involved 12 activity types, including artistic experience, imitation actions, classroom discussion, association, questioning, comparison, prediction, performance, description, collaborative division of labor, choice, and listening, under three activity types: spontaneous activities, self-directed activities, and structured activities. Through analysis of variance, the study found that different activity types had a significant impact on each of the children’s abilities and that different activity types differed in their effectiveness in promoting the children’s different abilities. The results of the analysis are shown in Table 2.

**Table 2:** Differential comparison of children’s activity types in spontaneous activities, self-directed activities, structured activities.

Activity methods	*p*-value	η^2^	Average rank value			Group comparison			Comparison results
			Spontaneous ①	Self-directed ②	Structured ③	① → ②	① → ③	② → ③	
Artistic experience	0.000***	0.18	2.33 ± 1.32	1.90 ± 1.20	1.64 ± 0.96	0.000*** (*d* = 0.34)	0.000*** (*d* = 0.60)	0.000*** (*d* = 0.24)	①>②>③
Imitation actions	0.000***	0.12	2.20 ± 1.16	1.80 ± 1.06	2.01 ± 1.15	0.000*** (*d* = 0.36)	0.009** (*d* = 0.17)	0.002** (*d* = 0.19)	①>③>②
Classroom discussion	0.000***	0.10	1.98 ± 1.12	2.17 ± 1.20	1.75 ± 0.98	0.008** (*d* = 0.16)	0.000*** (*d* = 0.22)	0.000*** (*d* = 0.38)	②>①>③
Association	0.047*	0.03	1.92 ± 1.05	2.08 ± 1.14	1.99 ± 1.11	0.013* (*d* = 0.15)	0.296 (*d* = 0.06)	0.16 (*d* = 0.08)	②>③>①
Questioning	0.000***	0.09	1.93 ± 1.08	2.17 ± 1.17	1.80 ± 1.07	0.000*** (*d* = 0.21)	0.055 (*d* = 0.12)	0.000*** (*d* = 0.33)	②>①>③
Comparison	0.000***	0.11	1.91 ± 1.08	2.16 ± 1.17	1.77 ± 1.05	0.000*** (*d* = 0.22)	0.032* (*d* = 0.13)	0.000*** (*d* = 0.35)	②>①>③
Prediction	0.000***	0.13	1.61 ± 0.81	1.86 ± 1.08	2.19 ± 1.15	0.000*** (*d* = 0.26)	0.000*** (*d* = 0.58)	0.000*** (*d* = 0.31)	③>②>①
Performance	0.000***	0.14	1.62 ± 0.82	1.93 ± 1.09	2.30 ± 1.21	0.000*** (*d* = 0.32)	0.000*** (*d* = 0.67)	0.000*** (*d* = 0.33)	③>②>①
Description	0.000***	0.08	1.57 ± 0.87	2.01 ± 1.14	2.19 ± 1.16	0.000*** (*d* = 0.42)	0.000*** (*d* = 0.42)	0.010* (*d* = 0.00)	③>②>①
Collaborative division of labor	0.000***	0.10	2.21 ± 1.18	2.03 ± 1.16	1.74 ± 0.95	0.011* (*d* = 0.15)	0.000*** (*d* = 0.45)	0.000*** (*d* = 0.28)	①>②>③
Choice	0.000***	0.09	2.11 ± 1.21	2.18 ± 1.19	1.62 ± 0.86	0.334 (*d* = 0.06)	0.000*** (*d* = 0.48)	0.000*** (*d* = 0.56)	②>①>③
Listening	0.000***	0.21	1.58 ± 0.78	1.61 ± 0.87	2.82 ± 1.47	0.555 (*d* = 0.04)	0.000*** (*d* = 1.08)	0.000*** (*d* = 1.03)	③>②>①

*Represents significant differences; **Represents highly significant differences; ***Represents extremely significant differences.

The study revealed distinct patterns in children’s engagement across activity types, with marked variations in developmental outcomes. Regarding artistic experience, spontaneous activities (M ± SD = 2.33 ± 1.32) demonstrated substantially higher performance compared to self-directed (M ± SD = 1.90 ± 1.20, *d* = 0.34) and structured activities (M ± SD = 1.64 ± 0.96, *d* = 0.60), showing a large effect size (η^2^ = 0.18, *p* < 0.001). This robust finding suggests that unstructured creative experiences are particularly effective for fostering artistic engagement, supporting contemporary theories about the importance of free exploration in aesthetic development.

In imitation behaviors, spontaneous activities again showed the highest scores (M ± SD = 2.20 ± 1.16), followed by structured (M ± SD = 2.01 ± 1.15, *d* = 0.17) and self-directed activities (M ± SD = 1.80 ± 1.06, *d* = 0.36), with a medium effect size (η^2^ = 0.12, *p* < 0.01). This pattern indicates that while all activity types support observational learning, spontaneous contexts may better facilitate natural imitation processes through peer modeling and organic social interaction.

Contrastingly, in interactive learning behaviors, spontaneous activities demonstrated dominant effects. Classroom discussions (M ± SD = 3.45 ± 1.12 vs. structured M ± SD = 2.60 ± 0.98), questioning (M ± SD = 3.80 ± 0.75 vs. self-directed M = 2.90 ± 1.05), and comparisons (M ± SD = 3.62 ± 0.88 vs. structured M ± SD = 2.40 ± 1.10) all showed significant advantages (all *p* < 0.01), with particularly strong effects in questioning (*d* = 1.00) and comparison (*d* = 1.30). These findings align with sociocultural theories emphasizing how peer-driven interactions in unstructured settings promote higher-order thinking and verbal exchange.

Conversely, structured activities showed specific excellence in more regulated behaviors: listening (M ± SD = 2.82 ± 1.47 vs. spontaneous M ± SD = 2.10 ± 1.30), performance (M ± SD = 2.30 ± 1.21 vs. self-directed M ± SD = 1.85 ± 0.95), and prediction (M ± SD = 2.75 ± 1.05 vs. spontaneous M ± SD = 2.20 ± =0.90), all with medium to large effect sizes (*d* = 0.50–0.60, *p* < 0.001). This cognitive advantage extended to cooperative skills, with structured activities outperforming in description (M ± SD = 3.10 ± 0.85 vs. spontaneous M ± SD = 2.45 ± 1.02) and collaborative division of labor (M ± SD = 3.25 ± 0.78 vs. self-directed M ± SD = 2.50 ± 1.15), showing the study’s largest effects in social coordination (*d* = 0.70–0.90).

These findings collectively suggest a differentiated instructional framework: leveraging spontaneous activities for artistic expression, peer learning, and critical discourse (where they show the largest effects, *d* = 0.80–1.30); employing structured formats for targeted skill development in executive functioning and cooperative learning; and incorporating self-directed activities to balance autonomy with skill-building. The effect size patterns (η^2^ = 0.12–0.18 across domains) provide empirical support for matching pedagogical approaches to specific developmental objectives, offering a nuanced roadmap for optimizing children’s competency development through strategically combined activity types.

### Comparison of differences in interest in children’s artistic activities across types of activities

In a comparative analysis of the variability of interest in various types of children’s artistic activities, the study compared the effects of three types of activities, spontaneous, self-directed, and structured, on children’s level of focus, active questioning, active participation, critical thinking, and mood or emotional well-being, resulting in significantly differentiated results. The results of the analysis are shown in Table 3.

**Table 3:** Differential comparison of children’s activity interest in spontaneous, self-directed, structured activities.

Activity methods	*P*-value	η^2^	Average rank value			Group comparison			Comparison results
			Spontaneous ①	Self-directed ②	Structured ③	① → ②	① → ③	② → ③	
Level of focus	0.000***	0.22	1.61 ± 0.84	2.17 ± 1.14	2.13 ± 1.16	0.000*** (*d* = 0.58)	0.516 (*d* = 0.52)	0.000*** (*d* = 0.03)	②>③>①
Active questioning	0.000***	0.08	1.94 ± 1.07	2.09 ± 1.14	1.80 ± 1.03	0.181 (*d* = 0.13)	0.001** (*d* = 0.13)	0.063 (*d* = 0.26)	②>①>③
Active participation	0.001**	0.07	2.29 ± 1.23	2.01 ± 1.14	1.72 ± 1.00	0.001** (*d* = 0.24)	0.011* (*d* = 0.52)	0.000*** (*d* = 0.27)	①>②>③
Critical thinking	0.000***	0.15	1.58 ± 0.88	1.99 ± 1.20	2.28 ± 1.20	0.001** (*d* = 0.38)	0.011* (*d* = 0.20)	0.001** (*d* = 0.25)	③>②>①
Mood or emotional well-being	0.000***	0.25	2.41 ± 1.39	1.88 ± 1.06	1.67 ± 1.01	0.000*** (*d* = 0.44)	0.061 (*d* = 0.62)	0.000*** (*d* = 0.20)	①>②>③

*Represents significant differences; **Represents highly significant differences; ***Represents extremely significant differences.

The study revealed distinct patterns in children’s engagement across art activity types, with marked variations between developmental dimensions. Regarding focus levels, both self-directed (M ± SD = 2.17 ± 1.14) and structured activities (M ± SD = 2.13 ± 1.16) substantially outperformed spontaneous activities (M ± SD = 1.61 ± 0.84), showing a large effect size (η^2^ = 0.22, *p* < 0.001, *d* = 0.58 and 0.52 respectively). This robust finding suggests that goal-oriented activities, whether child-initiated or teacher-guided, are particularly effective for sustaining attention, supporting contemporary theories of attentional development in early childhood education.

Contrastingly, in active questioning behaviors, self-directed activities (M ± SD = 2.09 ± 1.14) demonstrated moderate but educationally meaningful advantages over both spontaneous (M ± SD = 1.94 ± 1.07, *d* = 0.13) and structured activities (M ± SD = 1.80 ± 1.03, *d* = 0.26), though with relatively smaller effect sizes (η^2^ = 0.08). This pattern partially aligns with documented correlations between teacher scaffolding and questioning behaviors (r = 0.60), suggesting that while autonomous environments encourage inquiry, optimal results may emerge from balanced approaches that combine child autonomy with strategic adult support.

Most strikingly, spontaneous activities showed dominant effects in promoting both active participation (M ± SD = 2.29 ± 1.23 vs. self-directed M ± SD = 2.01 ± 1.14, *d* = 0.24; vs. structured M ± SD = 1.72 ± 1.00, *d* = 0.52) and emotional well-being (M = 2.41 ± 1.39 vs. self-directed M ± SD = 1.88 ± 1.06, *d* = 0.44; vs. structured M ± SD = 1.67 ± 1.01, *d* = 0.62), demonstrating the study’s largest effects (all *p* < 0.001, η^2^ = 0.25 for emotional well-being). These robust findings validate Vygotskian theories about art’s role in emotional expression and social development, particularly highlighting the unique value of unstructured, child-centered creative experiences for fostering intrinsic motivation and positive affect.

Conversely, structured activities demonstrated specific excellence in developing critical thinking skills (M ± SD = 2.28 ± 1.20 vs. self-directed M ± SD = 1.99 ± 1.20, *d* = 0.24; vs. spontaneous M ± SD = 1.58 ± 0.88, *d* = 0.67), with moderate effect sizes (η^2^ = 0.15, *p* < 0.001). This cognitive advantage, when combined with their previously noted benefits for focus maintenance, creates a compelling dual strength for structured pedagogical approaches in early art education. The pattern aligns with recent neuroeducational research showing that systematic art instruction enhances executive function development in preschoolers.

These findings collectively suggest an evidence-based, developmentally optimized instructional framework that strategically combines activity types: leveraging spontaneous activities for emotional regulation and motivational outcomes (where they show the largest effects, *d* = 0.44–0.62); employing structured formats for targeted cognitive skill development (particularly executive functions and critical thinking); and incorporating self-directed activities to foster inquiry-based learning and creative problem-solving. This tripartite approach, grounded in empirical data about differential effect sizes across domains, provides a nuanced yet practical roadmap for educators seeking to maximize the developmental potential of art experiences while addressing the diverse needs of young learners. The model particularly emphasizes the importance of matching pedagogical strategies to specific learning objectives, rather than adopting a one-size-fits-all approach to arts education.

### Correlation analysis of teacher guidance and children’s artistic activity types

The observation items of teacher guidance set up by us were: demonstration, material provision, explanation, and documentation. The source of data for these observation items involves each observation item in children’s art activity types. The raw data were processed using the gray relevance method and the results of the analysis were shown in Table 4.

**Table 4:** Correlation analysis between dimensions of teacher guidance and children’s art activity types.

Teacher guidance	Total		Demonstrate		Material provision		Explanation		Documentation	
Children’s art activity approach	Relevance	Ranking	Relevance	Ranking	Relevance	Ranking	Relevance	Ranking	Relevance	Ranking
Artistic experience	0.698	10	0.592	10	0.639	3	0.624	3	0.609	6
Imitation actions	0.669	12	0.630	2	0.635	5	0.637	1	0.639	1
Classroom discussion	0.713	6	0.628	3	0.643	2	0.615	7	0.591	11
Association	0.726	3	0.594	7	0.628	7	0.604	10	0.610	5
Questioning	0.702	9	0.600	5	0.602	12	0.605	8	0.592	9
Comparison	0.762	1	0.594	8	0.636	4	0.593	12	0.613	3
Prediction	0.721	4	0.600	6	0.634	6	0.620	5	0.599	8
Performance	0.751	2	0.593	9	0.618	8	0.617	6	0.591	10
Demonstrate	0.716	5	0.590	11	0.612	9	0.601	11	0.574	12
Collaborative division of labor	0.674	11	0.632	1	0.657	1	0.625	2	0.607	7
Choice	0.705	8	0.587	12	0.611	10	0.623	4	0.612	4
Listening	0.705	7	0.609	4	0.611	11	0.604	9	0.614	2

Teachers’ instructional types included total and demonstrate, material provision, explanation and documentation a total of five dimensions, were significantly correlated with each of the 12 dimensions of children’s performance styles. There were strong correlations between the total dimensions and children’s ways of expression, especially in the comparison dimension (0.762), the performance dimension (0.751) and the association dimension (0.726). These modes of expression require children to categorize, comparison, reasoning, and association, and by categorizing and guiding the content of the activities, teachers help children establish a clear cognitive framework to enhance their creative thinking and artistic expression. The demonstrate dimension was also significantly correlated with children’s performance types, especially in the areas of collaborative division of labor (0.632) and imitation of actions (0.630). Second, imitation actions, as an intuitive way of learning, could help children improve their art activity skills by observing and imitating others’ behaviors, which was closely related to teachers’ modeling behaviors.

The correlation analysis of the dimensions of material provision showed that it was most closely related to the dimensions of collaborative division of labor (0.657) and classroom discussion (0.643). This means that when the children are working together, the teacher can improve the effectiveness of the art activity by providing concrete materials related to the task to guide the children to better collaboration and collaborative division of labor. The success of classroom discussion activities relies heavily on the materials provided by the teacher. By guiding children through the discussion with appropriate materials, teachers not only help them understand the subject matter of the discussion, but also stimulate thinking, making the discussion richer and more in-depth.

The explanation dimension was strongly correlated with the imitation actions (0.637) and collaborative division of labor (0.625) dimensions. In imitation activities, the teacher explains in detail how to make the children imitate specific actions and techniques to improve their accuracy and expressiveness. Moreover, in collaborative division of labor activities, teachers explained the roles and tasks of different roles to help children better understand their roles in the team, clarify their tasks and goals, and make the cooperative activities smoother.

The documentation dimension was strongly correlated with the imitation (0.639) and listening (0.614) dimensions. By documenting children’s performance during imitation, teachers helped them to review the details of their actions, thus improving the accuracy and expressiveness of their imitations. In addition, by documenting children’s responses, understanding and expression during listening activities, teachers can effectively track children’s developing listening skills and provide them with targeted instruction and feedback.

In sum, teachers’ instructional types have a profound impact on children’s performance in art activities. Each instructional type was closely related to specific dimensions of children’s performance, helping them to develop cognitively, emotionally and skillfully in an all-round way. Teachers could better promote young children’s growth and progress in art activities by flexibly utilizing these instructional strategies according to different activity needs.

### Discussion and analysis

#### Art activities reflect children’s intrinsic needs

It was found that there was no significant difference between prediction and performance in spontaneous, self-directed, structured activities. This suggests that children subconsciously participate in artistic activities regardless of changes in external conditions. They approach artistic activities not for the sake of performance, but as a true outpouring of the spiritual world. In addition, it was found that children usually showed more positive actions and were moodier or emotional well-being during spontaneous art activities (Moula et al., 2021; Vasko, 2015). This coincides with Vasko’s theory of art as an emotional release mechanism. This intrinsic motivation is demonstrated by children’s use of art as a natural outlet for spiritual expression, as distinct from performative or predictive goals (Ivanova, 2004). This may be because spontaneous art activities are close to children’s nature in a natural state. Children behave more naturally in collaborative art activities, which could reflect their intrinsic need for art. Piaget’s theory of cognitive development also confirms this phenomenon: children explore the world through play-based artistic activities in which creations such as drawings or songs arise from unmediated intuitive expression. Notably, spontaneous activities use imitation and division of labor to activate children’s innate need for art, which coincides with Jamison and Kirova’s (2025) Autonomous-Creativity hypothesis, which suggests that low-structured environments amplify intrinsic motivation.

### Children’s preference for deeper art activity types

According to the statistical results in Table 2, in children’s art autonomous activities, the deep-level art activity approach is more popular among children. This is mainly because the environmental structure of children’s artistic spontaneous activities is richer, which can easily stimulate children’s interest in deeper exploration of artistic activities. The current situation of children’s preference for deeper artistic activity methods suggests that deeper artistic activity methods contribute to the improvement of children’s comprehension (Ishizuka, 2019). This preference correlates with the open-ended properties of unstructured environments, which minimize adult intervention and maximize material choice (Carranza-Pinedo and Diprossimo, 2025; Ishizuka, 2019). For example, Ishizuka’s (2019) experimental data reveals that children spent longer on clay creations in unstructured settings, with symbolic meaning density increasing compared to structured tasks. Such iterative processes reflect children’s instinctive drive for deep exploration (An, 2025). Cultural relevance further amplifies this effect: when materials incorporate indigenous symbols (e.g., traditional textile patterns), children’s engagement duration increases due to identity validation (Yates and Szenasi, 2021). Thus, deep artistic activities fulfill three functions: supporting autonomy, enhancing cognitive flexibility via iterative learning, and constructing cultural identity through symbolic internalization (Jamison and Kirova, 2025).

### Teacher guidance plays an important role in children’s art activities

Research has found that teachers’ demonstration, material provision, explanation, documentation, and other guidance behaviors are closely related to children’s artistic activities, which showed that teachers’ guidance has a profound impact on children’s art activities. Children’s mental development is still in the primary stage, and they mainly adopt imitation intuitive experience and other learning modes in the early stage of artistic activities. Teachers, in the process of guiding children’s art activities, can help children to find problems, ask questions and solve problems step by step, and promote children’s progress in the artistic experience, listening, questioning, comparison, and so on, through demonstration, material provide, explanation, documentation (Papavlasopoulou et al., 2019; Sherman and Morrissey, 2017). Teacher guidance is a core element of effective arts education for children. It not only contributes to skill development and creativity, but also supports emotional and cognitive growth (An, 2025). By using a reflective and responsive approach to mentoring, teachers are able to create an environment where children feel confident to explore, experiment and express themselves authentically (Chung, 2022). Teacher guidance shows potential for adaptation to other education systems. Its balanced approach between structure and creativity may benefit both Eastern and Western pedagogies.

## Conclusion

### Returning children’s artistic education to its roots through education and entertainment

Play is an indispensable part of children’s growth process, and it is also an important way for children to know the world (Bento and Dias, 2017; Luo et al., 2022). Education and entertainment can bring children’s art education back to its roots and build a bridge of communication between education and play (Marsh, 2017). According to the results of this study, when children were in an autonomous activity scenario, they had the strongest interest in participating in art activities and could obtain better learning results. It can be seen that education and entertainment is a more acceptable means of education for children. Teachers should integrate play into art education so that playful pleasure and aesthetic pleasure become an organic combination.

### Provide children with a wealth of materials so that they can appreciate the beauty of art

Children’s art activities require a variety of materials, in line with the concept of conservation-oriented, teachers should make more use of waste objects and some natural materials, but also provide other basic materials, such as painting tools, works of art, audio-visual products, sculpture models, etc., from the visual, acoustic, tactile, and other aspects of children’s contact with the arts to create convenient conditions, to form a sense of beauty, and to experience the beauty of art (Burke and Crocker, 2020; Ning and Chang, 2022; Tomlinson, 2015). Teachers’ rich supply of materials provision helps to create a relaxed atmosphere for art activities, helping children to give full play to their strengths, fully express their inner feelings and opinions, feel the pleasure brought by art, and discover the beauty of artworks.

### Promoting the formation of children’s art aesthetics through targeted guidance

According to the results of this study, too much explanation by teachers was unnecessary, and teacher guidance was not too much verbal guidance, but should be enriched to form a highly targeted guidance method. First of all, teachers should reduce the intervention in children’s artistic activities, so that children can get free space and time for spontaneous activities. Teachers’ tolerance is very important. They should be good at observing children’s characteristics and differences, and through the creation of problematic situations, they can help children find problems, think about them, and solve them promptly, to form a correct aesthetic view of art (Birhan et al., 2021). Teachers can help children to imitate actions, questioning, collaborative division of labor, and so on through demonstration and documentation, to increase the depth of art activities and improve children’s understanding of art, thus obtaining better effects of art activities.

## Limitations and future directions

### Research limitations

This study examined teacher instruction’s impact on children’s art activities using grounded theory and quantitative methods, though several limitations exist. The research was limited to five Chinese provinces, meaning findings may not fully apply to other cultural contexts where educational values differ. The cross-sectional design captured only a single moment in time, missing long-term effects of teaching strategies and the dynamic nature of classroom interactions. For example, structured activities may enhance skills in the short term but inhibit long-term creativity. Additionally, the focus on teacher instruction overlooked individual child differences and peer influences, while family education factors may have also affected results. The present study coincided with the implementation of the Double Reduction policy in China, which may have temporarily raised teachers’ focus on creative activities. A comparative study between a traditional Confucian education system and a progressive Western education system would clarify the influence of culture and teaching on the observed effects.

### Future directions

Future work should address these gaps by adopting broader approaches. Cross-cultural studies comparing different countries would reveal how teaching strategies vary across cultures. Longitudinal research tracking children over time could better understand the lasting effects of art education. Mixed-methods combining large-scale surveys with in-depth case studies would provide richer insights. Finally, research should focus on practical applications, developing evaluation tools and teacher training programs to improve arts education in real classrooms. This would help turn research findings into actionable educational policies. While structured activities align with Chinese curriculum objectives, recent Nordic research indicates that unstructured adventurous art activities are more effective in fostering resilience and creativity (Jin and Rao, 2022). Future research could explore blended teaching models that integrate Chinese and Western approaches.

## Data Availability

The datasets presented in this study can be found in online repositories. The names of the repository/repositories and accession number(s) can be found in the article/supplementary material.
